# Changes in the Infrared Thermographic Response of the Triceps Suralis Muscle During Ankle Flexion–Extension Until Exhaustion in Healthy Adults

**DOI:** 10.3390/sports13110383

**Published:** 2025-11-04

**Authors:** Alessio Cabizosu, Alessandro Zoffoli, Francisco Javier Martínez-Noguera

**Affiliations:** 1 Thermhesc Group, Chair of Ribera Hospital de Molina, San Antonio Catholic University of Murcia, Guadalupe, 30107 Murcia, Spain; acabizosu@ucam.edu (A.C.); azoffoli@alu.ucam.edu (A.Z.); 2 Research Center for High Performance Sport, Catholic University of Murcia (UCAM), Guadalupe, 30107 Murcia, Spain

**Keywords:** thermography, muscular fatigue, body temperature regulation, sex

## Abstract

Background: Infrared thermography and acute muscle fatigue are often correlated in sports medicine for assessing muscle health during and after exercise, but there are no known studies describing the response throughout exercise; therefore, this study aims to observe the variation in skin temperature (Tsk) during the execution of a maximal muscle fatigue protocol using concentric exercises in the triceps suralis. Methods: An open cross-sectional descriptive observational study of the posterior region of the leg (triceps suralis) was performed using 98 healthy subjects. Volunteers were subjected to a maximal fatigue protocol, with thermographic images analyzed at 25%, 50%, 75%, and 100% cumulative maximal fatigue. Results: Results showed a significant difference in time (*p* = 0.039; η^2^*p* = 0.026) and side (*p* =≤ 0.001; η^2^*p* = 0.120). Tukey’s post hoc analysis detected a significant difference in Tsk B between the R and L side (R, 30.3 ± 1.39 °C; L, 30.2 ± 1.39 °C), in Tsk at 25% (R, 30.2 ± 1. 27 °C; L; 30.1 ± 1.27 °C), in Tsk at 50% (R, 30.2 ± 1.29 °C; L, 30.1 ± 1.29 °C), and in Tsk at 75% (R, 30.3 ± 1.33 °C; L, 30.2 ± 1.33 °C). Conclusions: The results observed in this study show that infrared thermography is a valid tool for the measurement, analysis, and quantification of the tissue metabolic response of the muscular system, pre, during, and after exercise. Therefore, we believe that, for future studies, it would be interesting to find relationships between Tsk variations and other performance and metabolic variables. The focus on a single muscle and not directly measuring muscle activity are two limitations of this study.

## 1. Introduction

Infrared thermography (IRT) and acute muscle fatigue (until exhaustion) are correlated in the context of sports medicine in order to assess muscle health and monitor the body’s response to intense physical exertion by detecting thermal signatures associated with underlying physiological processes [1]. It is known that during the contractile process leading to muscle fatigue, biochemical, metabolic, and physiological changes are experienced in the muscle, such as increased lactate production [2], reduced energy levels (e.g., ATP) [3], and accumulation of metabolic by-products. These processes, in turn, generate alterations in vascular dynamics which can modulate peripheral blood flow and consequently, surface skin temperature [4].

In this context, according to some authors, IRT has been suggested in the last decade as a very efficient tool for monitoring the metabolic responses associated with muscle contraction, since this technique makes it possible to determine, from superficial skin analysis, changes in blood flow and physiological activity in deep tissues quickly and non-invasively, without contact [5,6]. IRT enables the identification of both hypothermic and hyperthermic patterns suggestive of persistent physiological stress, since cutaneous temperature reflects thermoregulatory adjustments connected to cellular activity in deeper layers [7].

In the neurological field, IRT is being used with very promising results in the quantification of trophic tissue in relation to functional muscle activity [8]. In the oncological field, the study of cancerous processes through thermography has advanced greatly over the last decade, providing information on the metabolic activity of tumor masses [9], and in the field of diagnostic medicine, there are numerous studies in which its reliability and validity have been proven in relation to different pathologies [10].

In the current landscape of sports medicine, this tool is being widely used, not only to detect early and late changes in muscle responses in relation to fatigue [11], but also to generate new knowledge through the assessment by thermograms of muscle overload and the prevention of injury or recovery from it in the medium to long term, especially in the lower extremities [12]. However, despite the new knowledge generated, there is still no single consensus on the specific thermoregulatory response obtained according to the type of exercise performed, the duration of the test, and the patient profile measured.

Some authors have advanced the hypothesis that in aerobic exercise, skin temperature (Tsk) tends to decrease in the exercised regions [13,14], while in strength training, a hyperthermic response is generated in the requested muscle groups [15,16]; however, there is a lack of studies confirming these results, and the literature often offers contrasting data [17,18]. This could be because current studies, on the one hand, have not reached a consensus on an exhaustive explanation from the thermographic point of view in relation to metabolic processes, and on the other, the samples are usually small and difficult to compare. Previous research examining thermographic responses to exercise was often limited by small sample sizes and the exclusive use of pre- and post-exercise measurements, without continuous monitoring throughout the exercise session. For example, Fink et al. [19] assessed changes in skin temperature before and after exercise, which limited their ability to capture dynamic thermoregulatory adjustments during the development of muscle fatigue.

Few studies have conducted continuous monitoring during exercise, a limitation that has made it difficult to understand dynamic thermoregulatory adjustments in real time. By contrast, our study incorporated continuous thermographic monitoring during the exercise protocol, allowing for real-time assessment of temperature fluctuations at different intensity stages (25%, 50%, 75%, and 100% of total effort). This methodological approach provides a more comprehensive understanding of thermal behavior during progressive fatigue, overcoming the limitations of previous studies that focused solely on static pre- and post-comparisons. Therefore, the present study aimed to investigate skin temperature variations during the execution of a maximal muscle fatigue protocol, using concentric exercises targeting the triceps suralis. The analysis also considered the participants’ genders and any potential distinctions between the left and right sides. We postulated that the protocol’s increasing metabolic demands would cause detectable temperature changes in the exercised area, indicating the degree of physiological stress.

## 2. Materials and Methods

### 2.1. Study Design

This open cross-sectional descriptive observational study of the posterior leg region (triceps suralis) in healthy subjects was performed in two laboratory views. The first familiarization session was performed 3 days before the development of the stress test and was used to confirm that the volunteer was suitable to participate in the study and to record data regarding age, sex, and BMI. From the familiarization day until the test days, participants were not allowed to do any type of exercise. In addition, in this session, the volunteers were familiarized with the thermographic evaluation position and the development of the maximal gastrocnemius fatigue protocol. During this phase, all volunteers were notified about exercise habits throughout the data-gathering timeframe. Considering the sensitivity and ongoing utilization of the calf muscles, it was recommended to decrease walking frequency and to refrain from standing for extended durations. The protocol used during the test is described in Section 2.4. The second session was used for measurement and stress testing. The study was conducted based on the Strengthening the Reporting of Observational Studies in Epidemiology (STROBE) guidelines [20]. As it enables the description and analysis of physiological and thermographic variables in a particular population under carefully monitored experimental conditions—without modifying any independent variables—this design was selected. The study’s goal of describing the skin temperature response during an exercise regimen until exhaustion is in line with the open cross-sectional descriptive observational approach.

All participants received an information sheet and signed the informed consent form at the first visit. The study was carried out in accordance with the Declaration of Helsinki for Research on Human Subjects [21] and was approved by the ethics committee of the Universidad Católica San Antonio de Murcia with code (CE032402).

### 2.2. Participants

None of the participants received economic incentives to participate in the study. A total of 100 healthy young subjects were recruited through an open advertisement (by email) at the Universidad Católica San Antonio de Murcia. Subjects who were of legal age, without gender or race limits, who were not semi-professional, professional or elite athletes, were included; subjects who had contraindications to muscle fatigue or who presented any issue that influenced the IT results, according to the investigators, were excluded. Participants were recreationally active, defined as individuals who engaged in planned, structured physical activity of moderate to vigorous intensity, including aerobic and strength training exercises, at least three times per week for a minimum of 30 min per session. Subjects in a febrile state, i.e., subjects with muscle or joint pain in the posterior region of the leg, were excluded from the study. Finally, 2 participants were excluded due to non-specific knee pain, so that a total of 98 subjects were considered eligible to participate in the study. The sample size was calculated using G*Power (version 3.1) for a repeated measures ANOVA (within–between contrast), considering the time × sex interaction. The following assumptions were considered: effect size f = 0.10 (small effect), α = 0.05, power = 0.90, number of groups = two (gender), number of measures = 10 (time levels were multiplied by 2 to incorporate the left/right factor), correlation between repeated measures = 0.60, and non-sphericity correction ε = 1. With these parameters, the required sample size was N = 12, correlation between repeated measures = 0.60, and non-sphericity correction ε = 1. With these parameters, the required sample size was N = 82 subjects (≈41 per group). To compensate for possible dropouts, additional recruitment (15%) was planned, resulting in a total sample of 95 subjects.

### 2.3. Equipment and Thermographic Protocols

**Figure 1:**
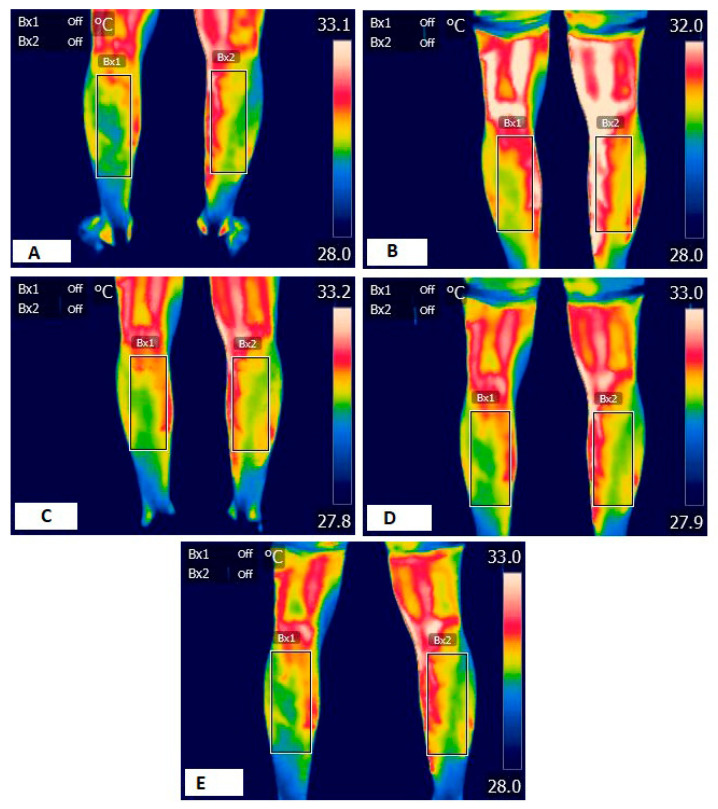
Thermographic sequence. (**A**) Basal thermography, (**B**) thermography 25%, (**C**) thermography 50%, (**D**) thermography 75%, and (**E**) final thermography, BX1: left leg; BX2: right leg.

The Flir E75 thermal camara (Wilsonville, OR, USA) was used, setting the emissivity at 0.98 [22]. BMI data were obtained using Tanita BC-545 Innerscan Segmental Body Composition Monitor (Tanita Corporation, Itabashi-ku, Tokyo, Japan). Volunteers underwent thermal acclimatization for 15 min in the room (25 m^2^) maintained at a constant temperature between 23 °C ± 1 °C, wearing shorts and short-sleeved T-shirts. The humidity never exceeded 40 ± 0.8%, and the atmospheric pressure was maintained at 1 ATM. The temperature and humidity in the room were monitored and recorded using a digital hygrometer and controlled using the air conditioning units located in the room. There were no electronic devices or sources of artificial light and heat near the measurement point that could influence the volunteers’ body temperatures. The thermographic device was turned on 1 h before the first recording and was located 1 m from the patient. The camera was positioned in front of the volunteers’ legs at an angle of 10°. To obtain a continuous thermographic follow-up during the test, photos were captured every five complete repetitions performed (ankle flexion–extension, full ROM). Once the total number of repetitions had been obtained, the images were subdivided into four quartiles, thus obtaining a baseline image (pre-protocol), one at 25% of maximum effort, another at 50% of maximum effort, another at 75% of maximum effort, and a final image at the end of the protocol (Figure 1). For example, if a volunteer performed 100 repetitions, since an image was recorded every 5 repetitions, the basal image, (T0) 0 repetitions; image 5 (T1), 25 repetitions; image 10 (T2), 50 repetitions; image 15 (T3), 75 repetitions; and image 20 (T4), 100 repetitions, were analyzed at the end of the protocol. In the case of patients who performed an odd number of repetitions, the image closest to the corresponding quartile was used. Image processing was performed in a blinded fashion by two investigators using Flir ResearchIR 4 software (Teledyne FLIR LLC, Wilsonville, OR, USA), as described by previous authors.

### 2.4. Maximum Fatigue Protocol

In the maximal gastrocnemius fatigue assessment, participants stood on a 25 cm elevated step with their knees extended, ensuring that the front part of their forefoot (head of the goal) contacted with the step. The midfoot and rearfoot were unencumbered to enhance maximum dorsiflexion and plantarflexion movement (ankle ROM). The patient was able to grip the stair rail with both hands to maintain balance, but not to lessen weight or move himself forward. Patients were required to maintain a constant speed of execution, (one second of the eccentric phase, dorsiflexion; and one second of the concentric phase, plantar flexion, was calculated using a digital stopwatch and verbally indicated by one of the researchers), performing the full range of motion of ankle dorsiflexion. If these requirements were not met, the test was terminated. A stress testing specialist observed that the range of motion and support were executed as described and assessed during the introductory session. The overall count of repetitions executed by each participant was recorded from the beginning of the test until the final possible repetition completed by the patients. The test was deemed complete when the volunteer was unable to execute further movements, the joint ranges were not adhered to, or the arms were utilized to aid the dorsiflexion phase. The area of the gastrocnemius was selected due to the extensive research previously completed in thermography linked to physical activity, along with the straightforwardness of conducting and evaluating both the physical and thermographic assessments. The participants completed the tests between 8:00 and 11:30 in the morning to minimize the effects of circadian rhythms on body temperature. Previous studies have demonstrated significant circadian rhythmicity in regards to skin temperature [23,24].

### 2.5. Statistical Analysis

IBM Social Sciences software (SPSS, v.21.0, Chicago, IL, USA) was used for statistical analysis. Data are presented as mean ± SD. The homogeneity and normality of the data were tested using the Levene and Shapiro–Wilk tests, respectively. To analyze intragroup and intergroup differences, a two-way repeated measures ANOVA was performed for Tsk, with a time factor (B vs. 25% vs. 50% vs. 75% vs. 100%) and a side factor (right (R) and left (L)), and sex, age, ethnicity, and dominant side as a factor between subjects, with BMI and number of total repetitions as covariates. A Tukey’s post hoc analysis was performed if significance was found in the ANOVA models. Partial eta squared (η^2^*p*) was also calculated as the effect size for the interaction of all variables in the ANOVA analysis. The following partial eta squared thresholds were used: <0.01, irrelevant; ≥0.01, small; ≥0.059, moderate; ≥0.138, large. The significance level was set at *p* ≤ 0.05.

## 3. Results

### 3.1. General Sample Description

Table 1 shows information about the general description of the sample. In comparison to the female participants (n = 50; 21.0 ± 2.47 years), the male participants (n = 48) were marginally older (22.7 ± 3.22 years). Additionally, the men’s body mass index (BMI) was higher (24.9 ± 4.02) than the women’s (22.7 ± 2.80).

The men’s baseline skin temperature values (right and left calf) were consistently higher than the women’s (29.5 ± 1.19 °C, right; 29.4 ± 1.16 °C, left) (31.2 ± 1.05 °C, right; 31.1 ± 1.05 °C, left, respectively). Throughout the various protocol intensities (25%, 50%, 75%, and 100% of total effort), this sex-related difference of roughly 1.5–1.7 °C remained constant. Women displayed somewhat lower values of ~29.3–29.5 °C throughout all stages, whereas men maintained constant values of about 31.0–31.1 °C.

Since both men and women maintained relatively stable Tsk values from 25% to 100% of exhaustion, indicating minimal acute thermal changes despite increased workload, no significant intra-sex variations were observed when taking intensity progression into account.

Lastly, although both groups showed high variability, men completed more repetitions until exhaustion (83.8 ± 49.4) than did women (76.3 ± 64.0).

### 3.2. Temperature

After performing two-way repeated measures ANOVA, at the calf Tsk level, a significant difference was found in time (*p* = 0.039; η^2^*p* = 0.026) and side (*p* =≤ 0.001; η^2^*p* = 0.120), along with a trend in the interaction of time x side (*p* = 0.068; η^2^*p* = 0.022).

**Figure 2:**
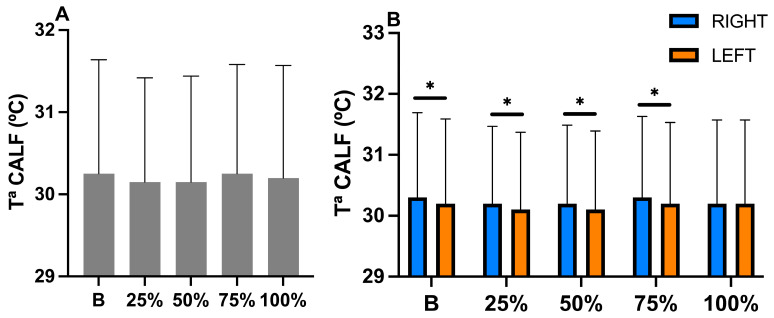
(**A**) Changes in calf temperature considering only time data during exercise until exhaustion. (**B**) Changes in calf temperature considering the interaction of time x side during exercise until exhaustion. * = significant differences between right and left side ≤ *p* = 0.05. T^a^ = temperature.

Post hoc Tukey analysis revealed that the right side (R) consistently exhibited slightly higher temperatures than did the left side (L) throughout the test. Post hoc analysis detected a significant difference in Tsk B between the R and L side (R, 30.3 ± 1.39 °C (IC 95% (30.1–30.6)); L, 30.2 ± 1.39 °C (IC 95% (30.0–30.5)); *p* = 0.016), in Tsk at 25% (R, 30.2 °C ± 1.27 °C (IC 95% (30.0–30.4)); L, 30.1 °C ± 1.27 °C (IC 95% (29.9–30.3)); *p* = 0.009), in Tsk at 50% (R, 30.2 °C ± 1.29 °C (IC 95% (30.0–30.5)); L, 30.1 °C ±1.29 °C (IC 95% (30.0–30.4)); *p* = 0.034), and in Tsk at 75% (R, 30.3 °C ± 1.33 °C (IC 95% (30.1–30.5)); L, 30.2 °C ± 1.33 °C (IC 95% (30.0–30.4)); *p* = 0.017) (Figure 2).

**Figure 3:**
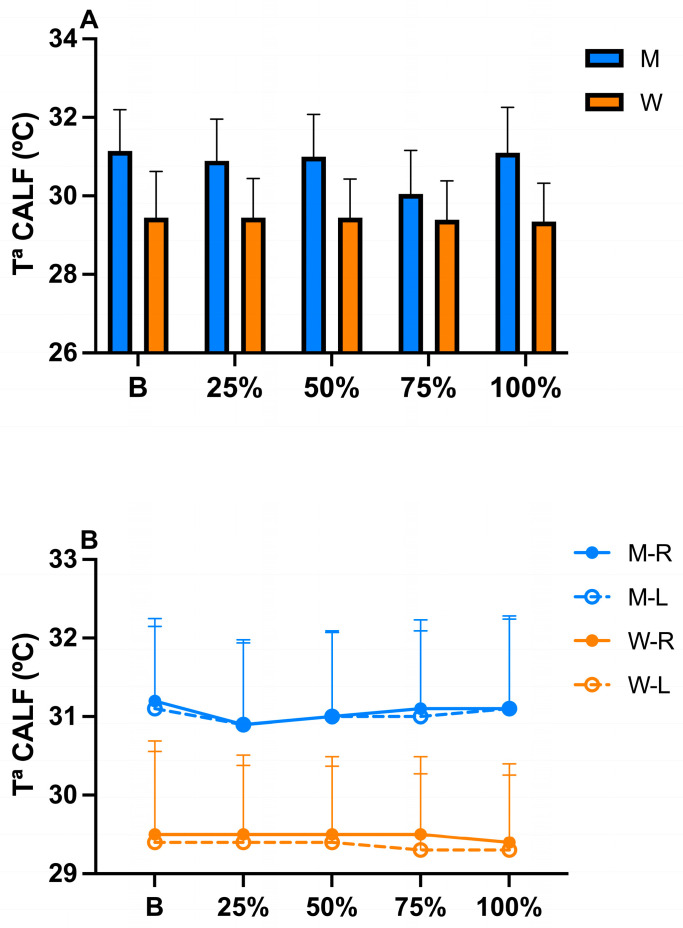
(**A**) Changes in calf temperature considering the time x sex interaction during exercise until exhaustion. (**B**) Changes in calf temperature considering time x side x sex interaction during exercise until exhaustion. M = men; W = women; R = right; L = left; T^a^ = temperature.

The ANOVA model detected no significant difference in the interaction time x sex (*p* = 0.110; η^2^*p* = 0.020), side x sex (*p* = 0.095; η^2^*p* = 0.029), and time x side x sex (*p* = 0.641; η^2^*p* = 0.007) (Figure 3), indicating that sex did not influence the temporal or lateral thermoregulatory responses under the present testing conditions.

## 4. Discussion

The aim of this study was to observe the variation in skin temperature during the execution of a maximal muscle fatigue protocol using concentric exercises on the triceps suralis. The results showed a significant difference in time (*p* = 0.039) and side (*p* =≤ 0.001). Tukey’s post hoc analysis detected a significant difference in Tsk B between the R and L side (R, 30.3 °C ± 1.39 °C; L, 30.2 °C ± 1.39 °C), in Tsk al 25% (R, 30.2 °C ± 1. 27 °C; L, 30.1 °C ± 1.27 °C), in Tsk al 50% (R, 30.2 °C ± 1.29 °C; L, 30.1 °C ± 1.29 °C), and in Tsk al 75% (R, 30.3 °C ± 1.33 °C; L, 30.2 °C ± 1.33 °C). The ANOVA model detected no significant differences in the interaction time × sex (*p* = 0.110), side × sex (*p* = 0.095), and time × side × sex (*p* = 0.641).

In relation to Tsk in basal conditions, despite showing significant differences between sides (*p* =≤ 0.001), it has been shown by other authors that only when such differences exceeded 0.6 °C between contralateral regions in the same anatomical region could they be considered as indicators of overload or pathological conditions, either superficial or deep [25,26]. Within this framework, our results are in agreement with those of previous studies in which, in healthy patients, in both other anatomical regions, as well as in twins, the temperature difference (ΔT) between extremities was never higher than 0.5 °C [8,27], representing a state of physiological normality of the tissues, ΔT ≤ 0.1 °C. Since these were healthy patients, factors that could influence these thermal asymmetries, such as inflammatory processes, vascular changes, or clinical situations, were therefore controlled and correctly ruled out.

Although descriptive analysis revealed minimal side-to-side differences (≤0.1 °C) in both sexes, the comparison between men and women showed a greater mean ΔT of approximately 0.7 °C (Figure 3B).

These findings confirm previous research in which it was observed that factors such as sex, age, training level, and BMI influence both the basal thermal response and the thermoregulatory response to fatigue [28,29,30]. As in our study, other authors showed that women tend to show different basal hypothermic patterns with respect to those of men [31] due to the concentration of muscle mass or fat tissue in certain areas, differences in the production of certain hormones, and distal vascular activity [32,33]. On the one hand, men exhibit a greater muscle mass, which generates more metabolic activity [31], and on the other, they carry less subcutaneous fat, which generates less thermal insulation, allowing greater heat transfer to the skin surface [34], and in addition, they display a lower tendency to peripheral vasoconstriction, which increases blood perfusion at distal structures [35]; thus, there is a certain consensus that men display higher skin temperatures than those of women, observable by IRT. Regarding the implication of body fat distribution, the higher percentage of fat in women and estrogen-mediated vasoconstrictive responses may limit heat transfer to the periphery [36]. In addition, gender differences have been observed in skin vascular conductance and autonomic control of thermoregulation during exercise and recovery [37], supporting the idea that thermographic patterns reflect underlying physiological differences between men and women.

The fact that men and women thermoregulate differently is of extreme importance in the clinical setting; in fact, for those physiotherapists who intend to design personalized thermal interventions and therapies on the basis of physical agents such as cold or heat, infrared thermography could be of great help to adjust treatment strategies and protocols according to individual thermal responses, improving, among others, the effects of the therapy. Future lines of research could explore the timing and delivery protocols of physical therapeutic agents aimed at increasing or decreasing muscle temperature as a function of gender.

In relation to Tsk throughout the protocol, a significant difference in time (*p* = 0.039) and side (*p* =≤ 0.001) of Tsk was described in this study. From a descriptive point of view, we can observe that, at 25% of the protocol, in men, a decrease in surface temperature of ΔT ≥ 0.2 °C is generated with respect to the basal temperature, while in women, the Tsk remains constant at ΔT ≥ 0.0 °C. These results could be evidence of a possible peripheral vasoconstriction that redirects blood flow toward more active muscles, as suggested by other authors [38,39,40], temporarily generating a reduction in vascular perfusion at the cutaneous level, which translates into a decrease in Tsk during exercise. However, unlike in the study by Ludwig et al. [38], in our study, it was only possible to observe this phenomenon in the male population, which leads us to believe that the thermoregulatory response in men does not follow the same dynamics as those in women. However, it should be noted that there are no known previous studies in which Tsk has been assessed during protocols according to sex, since most thermographic studies provide data from pre- and post-exertion. This opens a new window regarding the knowledge of the tissue metabolic response during exercise as a function of gender, of great interesting for personalizing program training sessions according to the physiological characteristics of each athlete.

At 50% of the protocol, in men, there was an increase in skin temperature that remained progressive and constant at 75% and until the end of the protocol, while in women the Tsk remained constant practically until the end of the protocol, where a decrease in temperature of 0.1 °C could be observed immediately after the end of the exercise. We know from other authors that, during exercise, due to the increase in muscle metabolism, an increase in heat release is generated and that part of this heat can be projected to the skin through the convection process, increasing the Tsk, which could explain the results obtained in this phase [41,42]. However, other authors showed that after intense sessions, a decrease in skin surface temperature has been observed due to the reduction in blood flow at the superficial level by migration to the deep level [5,43]. However, it should be noted that variations in Tsk depend mainly on the intensity, duration, and type of exercise, in combination with the characteristics of the anatomical region and the profile of the patient analyzed [44]. As in our work, some authors [45]) have observed increased Tsk in anaerobic training sessions, while others [46]) have observed hypothermic patterns after aerobic training; however, there are no known studies with a sample as large as ours in which this has been observed, analyzed, and described (using these parameters) throughout the protocol as a function of sex. Future studies could better clarify these novel findings found in our work.

Regarding the thermal variations found in our study pre-, during, and post-protocol, it should be noted that they are minimal compared to those observed in other works [41,42]. These differences could depend on the fact that the posterior region of the leg is a region too small to generate large thermoregulatory responses and that the type of exercise performed did not generate increased sweating or systemic response when compared to those noted in other studies in which larger anatomical regions that usually generated more robust metabolic changes were analyzed [47]. The small changes obtained in this study suggest that the exercise session did not promote acute injuries or important asymmetries, results consistent with an expected physiological response; in fact, the temperature differences between legs during and post-protocol were never higher than 0.2 °C. This study then presents some limitations, such as the anatomical region studied; a larger number of ROIs would have provided greater robustness to the results. However, it is worth mentioning that this study is the first work that analyzes the tissue metabolic response in such a large sample via IRT pre-, during, and post-exercise. In addition, since the sample size is substantial relative to those used in other research, our findings offer a clearer insight into the fundamental processes of skin temperature variations during physical activity.

Future research could expand the use of infrared thermography (IRT) by exploring other muscle groups and different types of exercise to better characterize thermal regulation patterns and their relationship to specific fatigue. IRT could also be evaluated as a non-invasive tool for monitoring the onset of fatigue and recovery in athletes, contributing to the individualization of training programs and injury prevention. Finally, combining IRT with other physiological measures, such as electromyography, blood lactate levels, or heart rate, would provide a more comprehensive view of muscle function and fatigue, improving the interpretation of thermographic changes and their practical relevance to athletic performance.

### Limitations and Strengths

This study has several limitations. First, the analysis was limited to the leg’s posterior region; adding more regions of interest (ROI) would have strengthened the conclusions. Second, IRT does not directly measure deeper muscle activity, even though it is very sensitive to surface temperature changes. This could be addressed by combining other measurements, like electromyography or near-infrared spectroscopy.

Despite these limitations, the current work offers some notable strengths. It is among the first studies to analyze sex-specific responses in a sizable sample while continuously monitoring Tsk during a maximal fatigue protocol. Given that female populations are frequently underrepresented in exercise physiology and thermography research, the inclusion of both men and women is a significant contribution. This study offers new evidence to better understand thermoregulatory processes during exercise by analyzing bilateral differences and incorporating sex-specific analysis. This knowledge could have applications in sports performance, injury prevention, and customized rehabilitation strategies.

## 5. Conclusions

The results observed in this study evidence that infrared thermography is a valid tool for the measurement, analysis, and quantification of the tissue metabolic response of the muscular system before, during, and after exercise, showing the changes in skin temperature throughout the maximum muscle fatigue protocol. However, due to the study design, it was not possible to compare these thermal changes with serum values or biomarkers. Therefore, we believe that for future studies, it would be interesting to find relationships between Tsk variations and other performance and metabolic variables. However, it should be noted that this technology does not directly measure deep muscle activity.

## Figures and Tables

**Table 1:** Data for age, body mass, height, BMI, and calf temperature during exercise protocol until exhaustion; mean (standard deviation).

	Men (n = 48)	Women (n = 50)
Age (years)	22.7 (3.22)	21.0 (2.47)
Body mass	79.3 (13.1)	64.2 (8.20)
Height	178.5 (4.1)	168.1 (3.60)
BMI	24.9 (4.02)	22.7 (2.80)
Tsk basal right (°C)	31.2 (1.05)	29.5 (1.19)
Tsk basal left (°C)	31.1 (1.05)	29.4 (1.16)
Tsk 25% right (°C)	30.9 (1.08)	29.5 (1.01)
Tsk 25% left (°C)	30.9 (1.040)	29.4 (0.980)
Tsk 50% right (°C)	31.0 (1.090)	29.5 (0.992)
Tsk 50% left (°C)	31.0 (1.070)	29.4 (0.970)
Tsk 75% right (°C)	31.1 (1.130)	29.5 (0.991)
Tsk 75% left (°C)	31.0 (1.090)	29.3 (0.971)
Tsk 100% right (°C)	31.1 (1.180)	29.4 (0.998)
Tsk 100% left (°C)	31.1 (1.140)	29.3 (0.954)
Total number of repetitions	83.8 (49.4)	76.3 (64.0)

BMI = body mass index; Tsk = skin temperature.

## Data Availability

The data presented in this study are available on request from the corresponding author due to privacy or ethical restrictions.

## Abbreviations

ATM	Atmosphere
BMI	Body Mass Index
ΔT	Difference in Temperature
IRT	Infrared Thermography
ROI	Region of Interest
TSK	Skin Temperature
STARD	Standards for the Reporting of Diagnostic Accuracy Studies
TISEM	Thermographic Imaging in Sports and Exercise Medicine
